# Efficient Preparation of pH-Sensitive Core–Shell Drug-Loaded Hydrogel Microcapsules and Their Application in Ulcerative Colitis Treatment

**DOI:** 10.3390/gels12080718

**Published:** 2026-08-13

**Authors:** Qingqing Xue, Yingli Li, Qing Ao, Guowang Chang, Yang Ji, Shizhang Chen, Ze Wang, Zifan Wang, Zhiqiang Li, Lei Zhao

**Affiliations:** 1 Key Laboratory of Biotechnology and Bioengineering of State Ethnic Affairs Commission, Biomedical Research Center, Engineering Research Center of Key Technology and Industrialization of Cell-Based Vaccine, Ministry of Education, Gansu Tech Innovation Center of Animal Cell, China-Malaysia National Joint Laboratory, Northwest Minzu University, Lanzhou 730030, China; y242530464@stu.xbmu.edu.cn (Q.X.);; 2 School of Bioengineering College, Northwest Minzu University, Lanzhou 730030, China; 3 College of Chemical Engineering, Northwest Minzu University, Lanzhou 730124, China; 4 School of Life Sciences and Engineering, Northwest Minzu University, Lanzhou 730124, China; 5 Department of Medical, Northwest Minzu University, Lanzhou 730030, China

**Keywords:** sodium alginate, carboxymethyl chitosan, core–shell microcapsules, pH sensitivity, olsalazine sodium, ulcerative colitis

## Abstract

Conventional microsphere drug carriers for ulcerative colitis (UC) face challenges such as limited residence time, variable drug release, and an increased risk of systemic exposure and side effects. In this study, pH-sensitive, core–shell hydrogel microcapsules were designed and fabricated using a BUCHI B-390 microsphere preparation device via electrostatic interactions and hydrogen bonds. Olsalazine sodium was encapsulated in the microcapsules, allowing for pH-responsive drug release in colon tissue for UC treatment in mice. XRD studies demonstrated the amorphous state of the drug in the formulation. The preparation of SCO microcapsules was optimized based on the drug encapsulation efficiency and the drug loading capacity, with the S_2_C_1_O microcapsule having the highest drug encapsulation efficiency (59.2%) and drug loading capacity (21.3%), and the production yield was approximately 62.5%. The degradation experiment results indicated that the alginate/CMCS hydrogel shell has anti-resistant and colon-targeted properties, with minimal drug leakage under acidic conditions (0.1% release at 2 h, pH 1.2) and rapid, controlled release at colonic pH (7.4) (cumulative release of 68.7% at 12 h), protecting the drug from gastric degradation. An in vivo experiment suggested that treatment with these microcapsules in UC mice significantly reduced inflammatory markers (NF-*κ*B p65 was reduced by 18.8% relative to the free drug group) and histological damage in UC models relative to free drug administration. The improved therapeutic efficacy is linked to precise localization in inflamed tissue, reducing systemic exposure and off-target effects. Overall, in vitro and in vivo studies demonstrated that this microcapsule system provides a promising alternative to existing UC drug delivery systems.

## 1. Introduction

Ulcerative colitis (UC) is a common chronic, recurrent inflammatory bowel disease with no specific cause [1,2]. In 2023, the global prevalence of UC was estimated to be approximately 5 million cases [3], with the number of cases increasing year after year [4]. Although the exact cause of the disease is unknown, it is believed to involve a complex interplay of factors, including genetic predisposition, the microbiome, environmental stressors, and immune dysregulation [5,6,7]. Symptoms of UC typically include abdominal pain, diarrhea, tenesmus, fatigue, and rectal bleeding [8,9,10]. Chronic pain and discomfort caused by UC significantly reduce the quality of life for the affected individuals compared with healthy populations [11]. Corticosteroids, aminosalicylates [12], antibiotics [13], immunosuppressants [14], and biologics [15] are currently used to induce and maintain remission in patients with UC, thereby preventing disease progression. Oral administration is the optimal strategy for UC treatment compared with alternative routes such as rectal delivery or intravenous injection, owing to its superior convenience, safety, and patient adherence [16,17]. However, because the colon lies at the distal end of the digestive tract, orally administered drugs must traverse the harsh gastrointestinal milieu prior to reaching the colon. This process frequently impairs drug stability and decreases bioavailability due to variable pH values along the gastrointestinal tract [18,19].

Hydrogel-based materials represent highly efficient drug carriers for UC therapy, particularly those incorporating stimuli-responsive polymers (SRPs) [20]. Over recent decades, SRPs have undergone a transformative evolution from simple environment-sensitive materials (that are sensitive to temperature, pH, ionic strength, and light) [21] to sophisticated multi-functional architectures capable of autonomous therapeutic regulation [22]. In the context of colonic disease therapy, this evolution has been particularly impactful, as the gastrointestinal tract presents a steep pH gradient from highly acidic gastric conditions (pH ≈ 1.2) to the near-neutral colonic environment (pH ≈ 7.4) [23]. Consequently, intelligent biopolymer matrices have been increasingly engineered to exploit these pathophysiological signatures—pH-responsive core–shell hydrogel microspheres, for instance, maintain structural integrity under gastric acidic conditions to protect encapsulated therapeutics from premature degradation while selectively disintegrating in the colonic pH environment to achieve localized drug release [24,25].

Natural polysaccharides have been widely used as the pH-sensitive shell material of microcapsules due to their excellent properties, including biocompatibility, biodegradability, non-toxicity, renewability, low cost, and processability [26]. Moreover, researchers have reported that polysaccharides can target the colon wall through electrostatic adsorption, hydrogen bonding, or the formation of disulfide bonds, which is beneficial to drug delivery [27]. Sodium alginate (SA) is a linear anionic polysaccharide derived from brown algae. It is composed of *α*-L-guluronic acid (G) and *β*-D-mannuronic acid (M) monomers linked together (1→4) [28], which are rich in carboxyl groups. Under acidic conditions, these carboxyl groups are protonated, limiting drug release. Under alkaline conditions, they deprotonate, resulting in strong pH sensitivity [29]. Its excellent gel-forming properties and pH sensitivity make it an ideal polymer for oral drug delivery systems for UC treatment [30,31]. However, the most common disadvantage of SA-based carriers is their high porosity, which makes them unstable at high pH levels and causes premature drug release [32,33]. When complexed with cationic polymers—such as chitosan or poly-L-lysine via electrostatic assembly—the pore architecture becomes tunable through modulation of the stoichiometric ratio between anionic carboxymethyl (–COO^−^) and cationic (–NH_3_^+^) groups [34]. Therefore, SA has been modified or combined with other polymers to create polyelectrolyte composites to overcome this limitation [35,36,37]. Carboxymethyl chitosan (CMCS) is a key chitosan derivative, which has cationic amino groups and anionic carboxyl groups [38,39,40]. At higher pH, acidic groups are ionized, so CMCS swells and has significant pH sensitivity, facilitating drug delivery at elevated pH levels [41]. Therefore, SA and CMCS can be combined as the pH-sensitive carrier material. In previous research, SA/CMCS hydrogels were crosslinked with chemical crosslinkers, such as genipin [42] and glutaraldehyde [43], and used as oral drug delivery systems. However, the use of chemical crosslinkers may result in undesirable drug interactions as well as toxic side effects from residual crosslinkers [44].

Moreover, SA- and CMCS-based drug delivery systems are mostly generated by directly combining the carrier materials and pharmaceuticals or by adsorbing medications onto the carriers. Jing et al. [45] created hydrogel beads by combining SA, CMCS, and lactoferrin, which could be used as carriers in oral protein delivery systems, allowing therapeutic proteins to be transported and released in the intestine. Xie et al. [46] created a gel made of SA and CMCS that delivered medicines by adsorbing bovine serum albumin. They recreated gastrointestinal conditions for in vitro release testing. However, these methods produce simple drug-carrier composites, which result in the unequal distribution of hydrophilic/hydrophobic medicines and uneven encapsulation efficiencies. Furthermore, they may cause premature drug leakage during storage and increase the risk of elevated local drug concentrations caused by burst release during in vivo delivery.

Microencapsulation technology provides an effective solution by creating a “core–shell” structure that physically isolates the medicines within the carrier’s core while the shell material serves as a pH-sensitive barrier. This structure not only eliminates interactions between medications and carriers but also improves the uniform distribution and encapsulation stability of hydrophilic and hydrophobic pharmaceuticals. Furthermore, by controlling the shell thickness, porosity, and degradation rates, it efficiently prevents leakage during storage and burst release following oral administration. This results in a smoother, more sustained drug release profile, which reduces local toxicity risks while improving the safety and therapeutic efficacy of oral delivery [47]. Emulsification crosslinking [48], in situ polymerization [49], and layer-by-layer self-assembly [50] are some of the most common microcapsule preparation procedures. With the above methods, the properties of the microcapsules cannot be efficiently optimized. However, combining co-extrusion technology with a microsphere preparation apparatus simplifies operation, resulting in capsules with adjustable and uniform sizes, excellent sealing integrity, and high drug loading capacity and encapsulation efficiency.

Herein, pH-responsive core–shell microcapsules were rapidly fabricated via a one-step co-extrusion strategy using a microencapsulation granulator; SA, CMCS, and citric acid (CA) were employed to construct carriers for targeted intestinal drug delivery. The microcapsules possessed a gel matrix core consisting of SA and CMCS loaded with olsalazine sodium (Olsa), which was enclosed using a composite shell fabricated from identical polymeric materials. After completing in vitro safety tests, the drug release profiles were characterized under simulated gastrointestinal environments. The anti-inflammatory performance and biological safety of the microcapsule system were further validated in a DSS-induced murine colitis model.

## 2. Results and Discussion

### 2.1. Design and Synthesis of the Microcapsules

**Figure 1:**
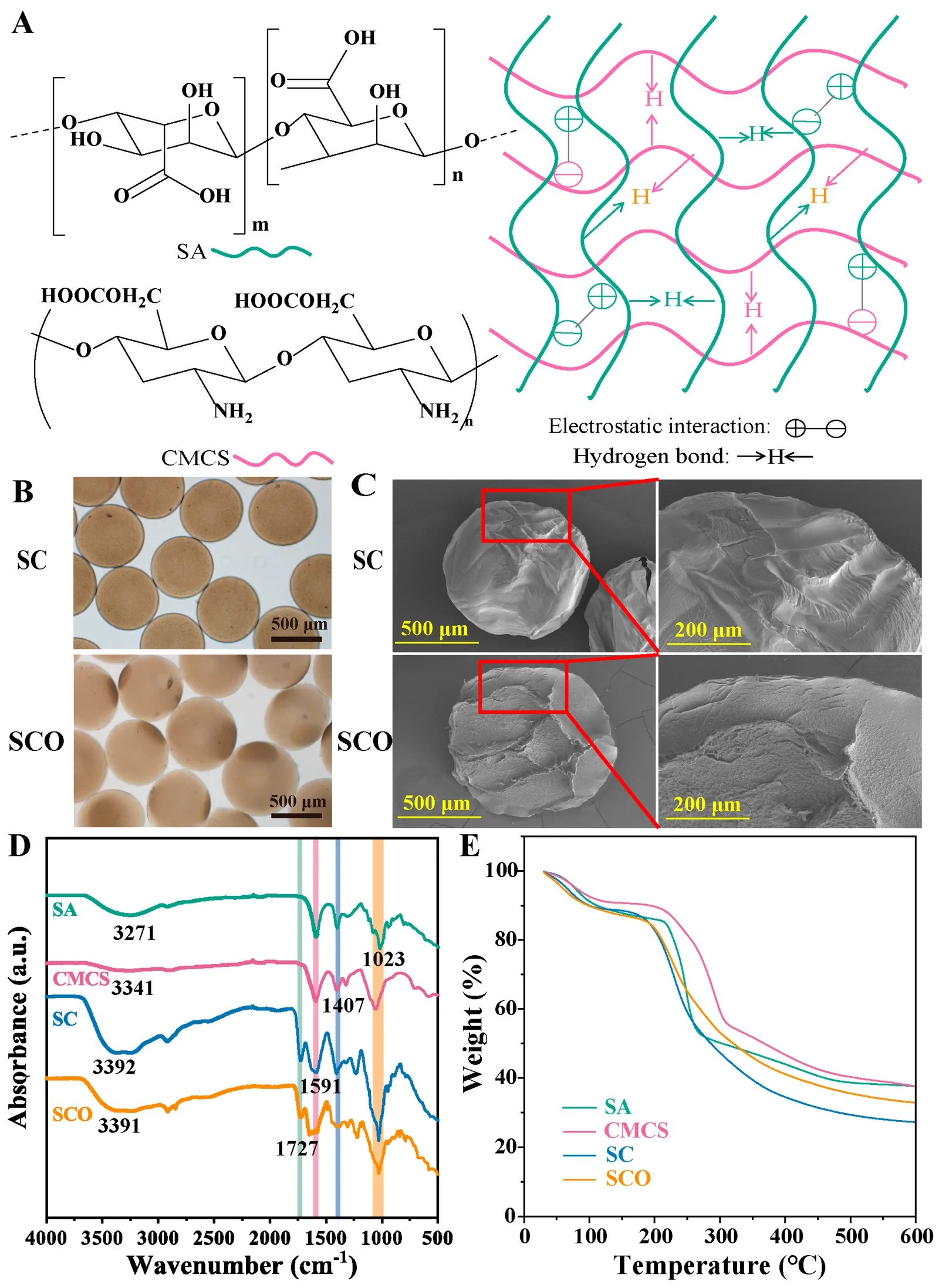
(**A**) Schematic illustration of the molecular structures of SA and CMCS, along with electrostatic interactions and hydrogen bonds formed between the two polymers inside SC microspheres and SCO microcapsules. (**B**) Bright-field optical microscope images of SC microspheres and core–shell SCO microcapsules (scale bar: 500 μm). (**C**) SEM images of SC microspheres and SCO microcapsules at two magnifications (scale bars: 500 and 200 μm). (**D**) FT-IR spectra of SA, CMCS, SC microspheres, and SCO microcapsules. (**E**) TGA analysis results of SA, CMCS, SC microspheres, and SCO microcapsules.

The p*K*a value of the amino group in CMCS is around 6.5, while that of the carboxyl group is around 2.7 [45]. The p*K*a value of alginate’s carboxyl group is around 3.5 [51,52]. In the acidic CA solution, most of the carboxyl groups in SA are protonated to –COOH, but some of the carboxyl groups in CMCS stay as –COO^-^. The amino groups of CMCS are almost fully protonated to –NH_3_^+^. Crosslinking is predominantly driven by hydrogen bonding interactions, with some crosslinks generated by electrostatic interactions [45,53] (as illustrated in Figure 1A).

The inverted microscopy images in Figure 1B reveal that SC and SCO particles prepared under identical fabrication conditions are micrometer-sized and spherical, and SCO exhibits a slightly larger particle size than SC; the core–shell structure of SCO microcapsules compared with SC microspheres has been highlighted in the Supporting Information (Figure S1). Moreover, in order to characterize the formation of the core–shell structure in SCO microcapsules, the fluorescence microscopy image result of the microcapsule is illustrated in Figure S2, which indicates the successful preparation of the core–shell structure of the SCO microcapsules. The freeze-dried SC and SCO have dimensions of 327.2 ± 105.1 and 469.1 ± 76.2 μm, respectively, with corresponding polydispersity indices (PDIs) of 0.103 and 0.026, respectively. These PDI values are comparable with those reported for similar extrusion-based microsphere systems (0.078–0.099) [54]. In comparison with SC microspheres, the introduction of Olsa increases the average particle size of the SCO microcapsules (Figure S3). Moreover, the SEM results illustrated that freeze-dried SC microspheres had mostly intact spherical shapes with wrinkles on the surface, which indicated that the polymer network had “uniformly shrunk” after drying. Following the addition of Olsa, the SCO microcapsules collapsed and became irregular in overall morphology (Figure 1C). These morphological differences are attributed to differential ice crystallization kinetics and distinct collapse temperatures between the dense chitosan–alginate polyelectrolyte complex shell and the hydrated alginate core. Such disparities generated thermomechanical stresses during fragmentation, ultimately resulting in an irregular morphology characterized by the coexistence of locally densified regions and loosely fragmented zones.

Figure 1D displays the FT-IR spectra of pure SA, CMCS, blank SC microspheres, and drug-loaded SCO microcapsules. For raw SA, characteristic peaks at 1407 and 1023 cm^−1^ corresponded to the symmetric stretching vibration of –COO^–^ and –C–O–C– glycosidic bond stretching of the polysaccharide skeleton [55,56], respectively. Raw CMCS showed similar peaks at 1407 and 1058 cm^−1^, attributed to carboxymethyl modification and pyranose ring vibration of the chitosan backbone. After electrostatic complexation and hydrogen bonding between SA and CMCS, the asymmetric stretching vibration peak of –COO^−^ (1591 cm^−1^) in both SC microspheres and SCO microcapsules exhibited an obvious shift, accompanied by the emergence of a characteristic peak at 1727 cm^−1^ (corresponding to the C=O stretching vibration of protonated –COOH). These changes were observed consistently in all formulations (blank SC microspheres and drug-loaded SCO microcapsules), confirming the uniform formation of polyelectrolyte complexes.

The above results indicated that the acidic environment of the CA solution partially protonated –COO^−^ groups into –COOH groups. These newly formed –COOH groups established strong hydrogen bond interactions with -OH groups present in CMCS and SA. Correspondingly, the broad absorption band around 3200~3500 cm^−1^ became wider and more intense in both SC microspheres and SCO microcapsules, directly verifying the enhanced hydrogen-bonding interactions between SA and CMCS [44,45,57]. The consistent peak shifts and spectral changes observed for all formulations confirmed the successful construction of the polymer matrix via electrostatic and hydrogen-bonding interactions.

TGA was further performed to investigate the thermal stability and compositional variation in pure SA, CMCS, SC microspheres, and SCO microcapsules (Figure 1E). All four samples exhibited an initial gradual weight loss below 200 °C, which was attributed to the evaporation of bound water and free water adsorbed in the hydrophilic polysaccharide networks of SA and CMCS [45,58]. Raw SA and CMCS exhibited their major thermal decomposition starting at approximately 200~220 °C, corresponding to the pyrolytic breakdown of the polysaccharide backbone, decarboxylation, and glycosidic bond cleavage of biopolymer chains. After electrostatic crosslinking to form SC microspheres, the decomposition trend clearly shifted; SC microspheres presented a lower residual weight than pure SA and CMCS across the whole heating range, confirming the formation of a polyelectrolyte complex that altered the original thermal degradation. Compared with SC microspheres, drug-loaded SCO microspheres showed a distinct intermediate weight loss stage at 220~320 °C, which originated from the thermal decomposition of encapsulated Olsa molecules. Meanwhile, the final char residue of SCO was higher than that of SC, further verifying successful drug incorporation into the core–shell carrier.

### 2.2. Optimization of the SCO Microcapsules

SC microspheres were created by combining SA and CMCS with a single-channel microencapsulation granulator. Olsa was chosen as the encapsulating medication because it produces 5-aminosalicylic acid (5-ASA) in the colon in response to luminal azoreductase, helping to reduce colonic inflammation and symptoms, promote colonic healing, and alleviate pain [59,60,61]. Figure S4 depicts Olsa’s standard release curve at various pH levels. Olsa was added to the SA/CMCS blend solution at a concentration of 30 mg/mL as the core, with different SA/CMCS ratios in the shell. Microcapsules were formed using a dual-channel microencapsulation granulator and were designed as S_3_C_1_O, S_2_C_1_O, S_1_C_1_O, S_1_C_2_O, and S_1_C_3_O. As illustrated in Figure S5, microcapsules prepared with different ratios of SA and CMCS had a similar surface morphology.

The FT-IR spectra of SCO microcapsules were also characterized, where obvious peak shifts were observed compared with their blank SC counterparts (Figure S6A). This indicated that the polymer interaction network remained stable after drug loading. Distinct differences in peak patterns and shift ranges were also found between different SCO groups, revealing that the polymer ratio could regulate the interaction strength between the matrices. TGA curves were applied to evaluate thermal stability (Figure S6b); pure SA and CMCS displayed obvious two-stage weight loss upon heating. Compared with blank SC microspheres, the thermal stability of SCO microcapsules was improved, while all SCO formulations exhibited similar thermal stability to one another (Figure S6B).

**Figure 2:**
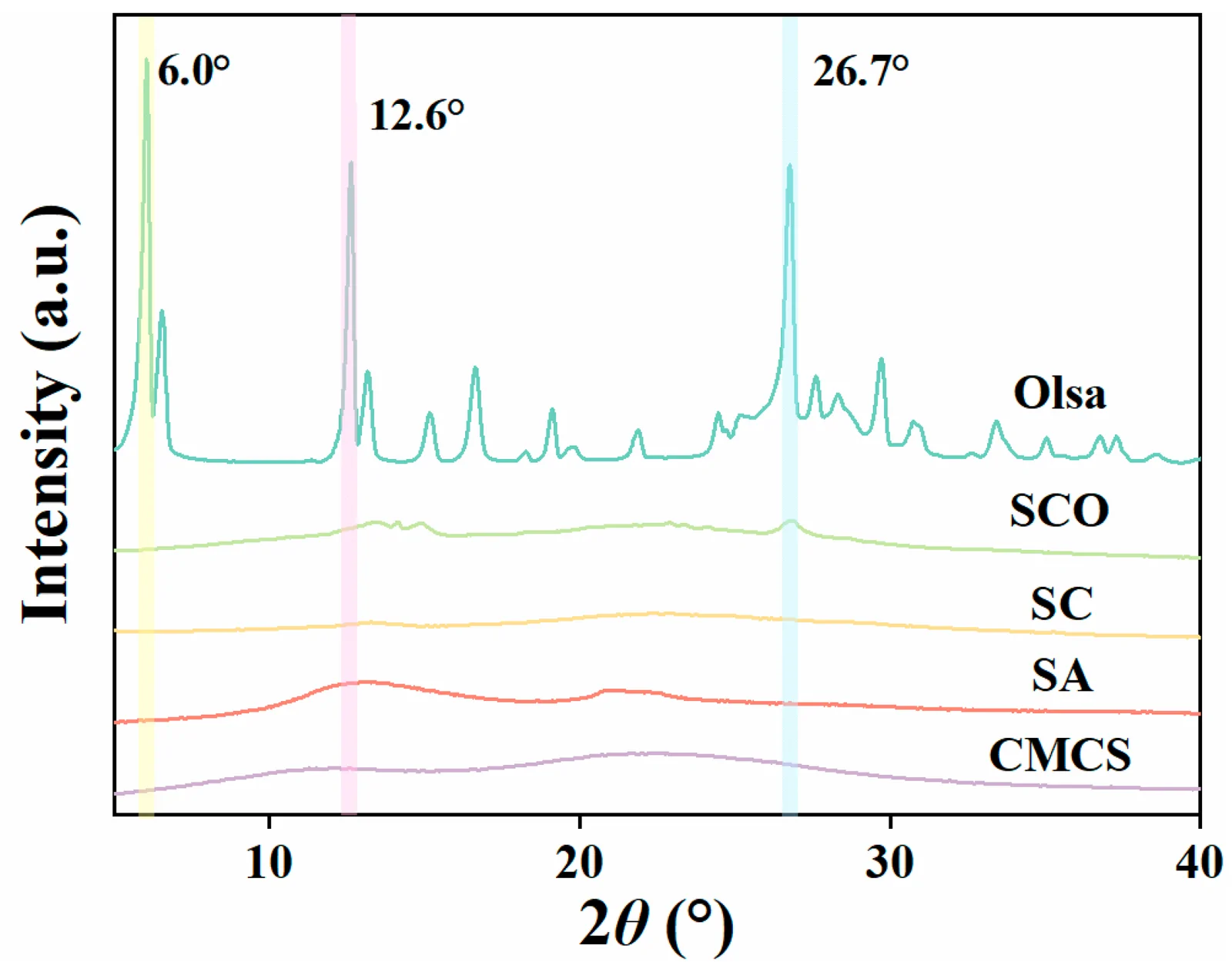
XRD curves of free olsalazine sodium (Olsa), carboxymethyl chitosan (CMCS), sodium alginate (SA), SC microspheres, and SCO microcapsules.

XRD was employed to investigate the crystalline properties of raw materials and as-prepared microcapsules. The XRD patterns of raw Olsa, SA, CMCS, SC microspheres, and drug-loaded SCO microcapsules are presented in Figure 2. Raw Olsa exhibited several sharp diffraction peaks at 2*θ* of 6.0°, 12.6°, and 26.7°, which confirmed its typical crystalline structure. In contrast, CMCS and SA merely exhibited broad, weak diffraction humps, indicating their predominantly amorphous nature. Blank SC microspheres retained the broad diffraction signals originating from the polymer matrix, with no observable crystalline peaks. Notably, the characteristic sharp peaks of crystalline Olsa completely vanished in the pattern of SCO microcapsules, with only the broad amorphous diffraction envelope of the polymeric carrier observed. This phenomenon demonstrated that Olsa was successfully incorporated into the microcapsule matrix in an amorphous or molecularly dispersed state rather than forming separate crystalline drug domains. 

**Figure 3:**
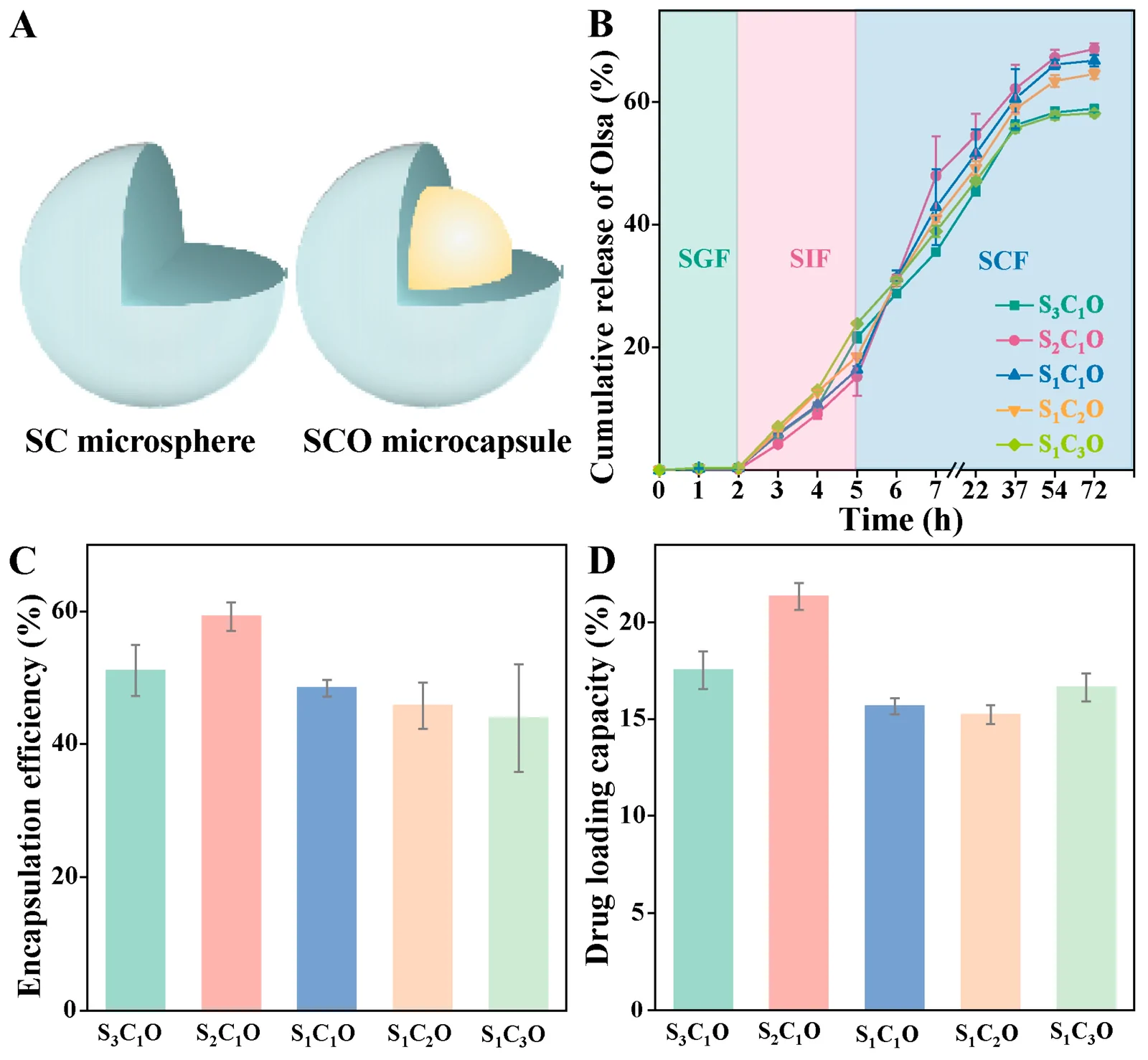
(**A**) Schematic illustration of the structure of SC microsphere and SCO microcapsule loaded with olsalazine. (**B**) In vitro cumulative drug release profiles under simulated gastric fluid (SGF, pH 1.2), simulated intestinal fluid (SIF, pH 6.8), and simulated colonic fluid (SCF, pH 7.4). (**C**) Drug encapsulation efficiency of SCO microcapsules prepared with different ratios of SA and CMCS (S_3_C_1_O, S_2_C_1_O, S_1_C_1_O, S_1_C_2_O, and S_1_C_3_O). (**D**) Drug loading capacity of SCO microcapsules prepared with different ratios of SA and CMCS (S_3_C_1_O, S_2_C_1_O, S_1_C_1_O, S_1_C_2_O, and S_1_C_3_O). All data are presented as mean ± standard deviation.

Then, the release properties of Olsa in these SCO microcapsules (Figure 3A) were assessed under simulated pH settings in various gastrointestinal segments. The release curves (Figure 3B) show pH-sensitive release behavior, with cumulative release increasing as the medium pH climbed. Under simulated stomach fluid (SGF, pH 1.2) conditions for the first 2 h, Olsa demonstrated negligible release across all formulations. Between 3 h and 5 h, under simulated intestinal fluid (SIF, pH 6.8) conditions, Olsa was released in all groups, with the total release nearing 20%, while S_2_C_1_O had the lowest release. Under simulated intestinal circumstances (SCF, pH 7.4), the amount of released Olsa increased dramatically and remained stable, decreasing systemic exposure and preventing unpleasant effects, while the release rate of Olsa in S_2_C_1_O was the highest. The result was consistent with the swelling ratio of SC microspheres prepared at various ratios of SA and CMCS (Figure S7), suggesting that the gel shell formed by SA and CMCS plays a decisive role in the microencapsulation effect.

Then, the encapsulation efficiency (EE) of Olsa in different SCO microcapsules was calculated and ranged from 43.9% to 59.2% (Figure 3C). The encapsulation efficiency of Olsa in the S_2_C_1_O microcapsule was the highest, reaching 59.2%. Drug loading (DL) was also calculated, which varied from 15.2% to 21.3% (Figure 3D) for different SCO microcapsules, and the value was also the highest for S_2_C_1_O microcapsules. Therefore, S_2_C_1_O microcapsules were selected for subsequent experiments. Moreover, the production efficiency of the freeze-dried S_2_C_1_O microcapsules was 62.5% (apparently 1.1 × 10^6^ microcapsules/s).

At last, the in vitro release kinetics of S_2_C_1_O microcapsules were investigated with different kinetic models, including Korsmeyer−Peppas, Higuchi model, Hixson−Crowell, first-order, and zero-order [62] (Figure S8 and Table 1). The Higuchi model showed the highest correlation coefficient (*R*^2^ = 0.817), followed by the first-order (*R*^2^ = 0.805), Korsmeyer−Peppas (*R*^2^ = 0.764), Hixson−Crowell (*R*^2^ = 0.714), and zero-order models (*R*^2^ = 0.637). The diffusion exponent *n* of the Korsmeyer−Peppas model was 0.51 (0.5 < *n* < 1) [62], indicating an anomalous no-Fickian transport mechanism. These results demonstrated that the Olsa released from the S_2_C_1_O hydrogel microcapsules was governed by the combined effect of drug diffusion and hydrogel matrix swelling, rather than polymer erosion.

### 2.3. Biocompatibility of the Microcapsules

**Figure 4:**
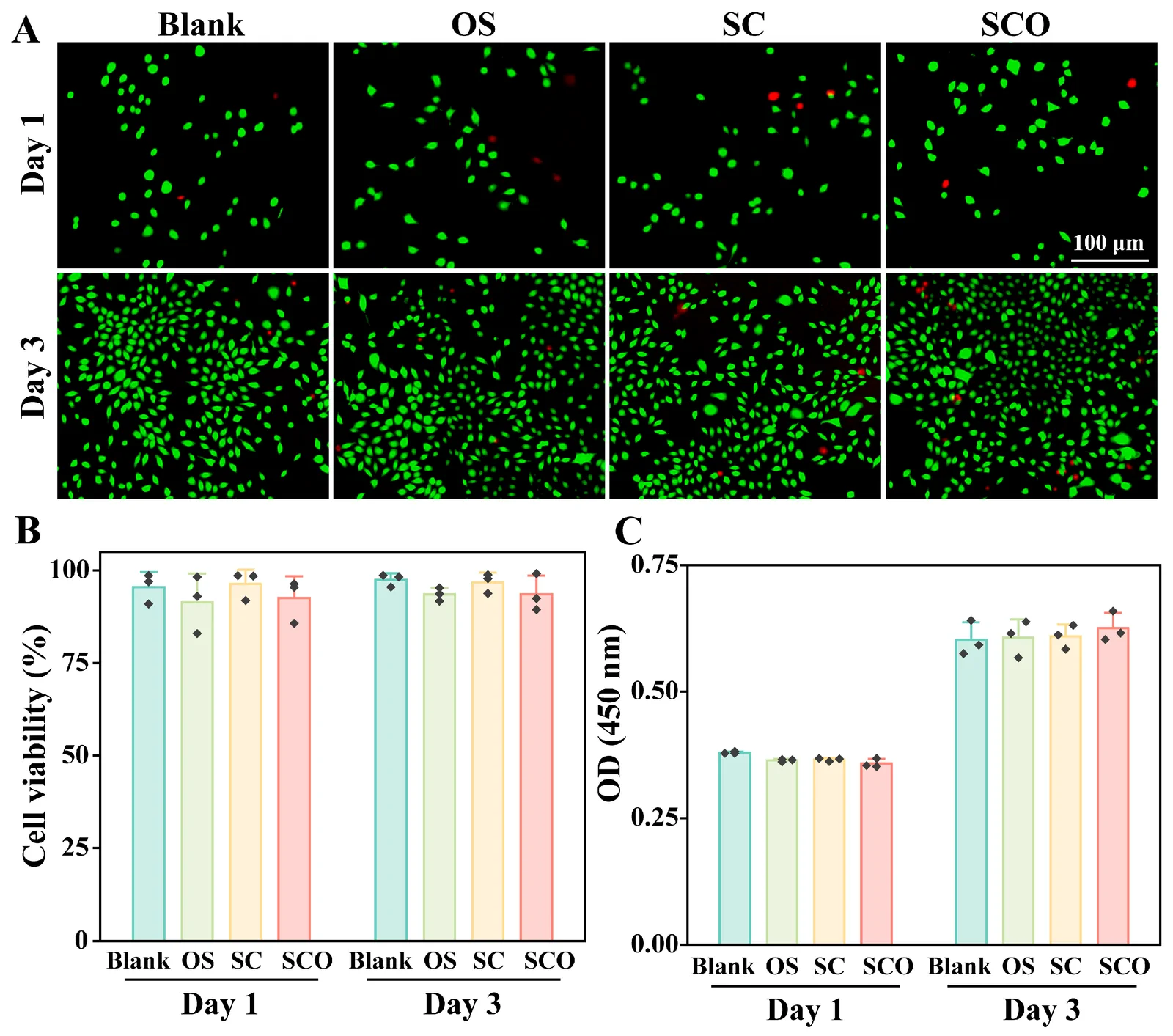
In vitro biocompatibility assessment of OS, SC microspheres, and SCO microcapsules using L929 fibroblast cells. (**A**) Representative live/dead fluorescence staining images of L929 cells after 1-day and 3-day incubation with different samples; live cells emit green fluorescence, dead cells emit red fluorescence, black squares represent different experimental groups, scale bar: 100 μm. (**B**) Quantitative statistical analysis of cell viability (%) on Day 1 and Day 3. (**C**) Cell proliferation measured via the CCK-8 assay by detecting absorbance at 450 nm. All data are presented as mean ± SD.

The biocompatibility of the microcapsules was assessed using cytotoxicity tests with mouse L929 fibroblasts, and cell compatibility was evaluated using live/dead cell labeling (Figure 4A). Across the groups, the results revealed plentiful green fluorescent dots (live cells) and few red fluorescent dots (dead cells). Compared with the control group, all experimental groups had a survival rate higher than 90% (Figure 4B), which revealed high cell viability. In addition, CCK-8 tests showed that L929 fibroblasts grew substantially in all groups from Day 1 to Day 3, with no obvious differences between the groups (Figure 4C). This validated that Olsa, SC microspheres, and SCO microcapsules have good cellular compatibility.

### 2.4. In Vivo Therapeutic Efficacy of SCO Microcapsules on DSS-Induced Colitis Mice

**Figure 5:**
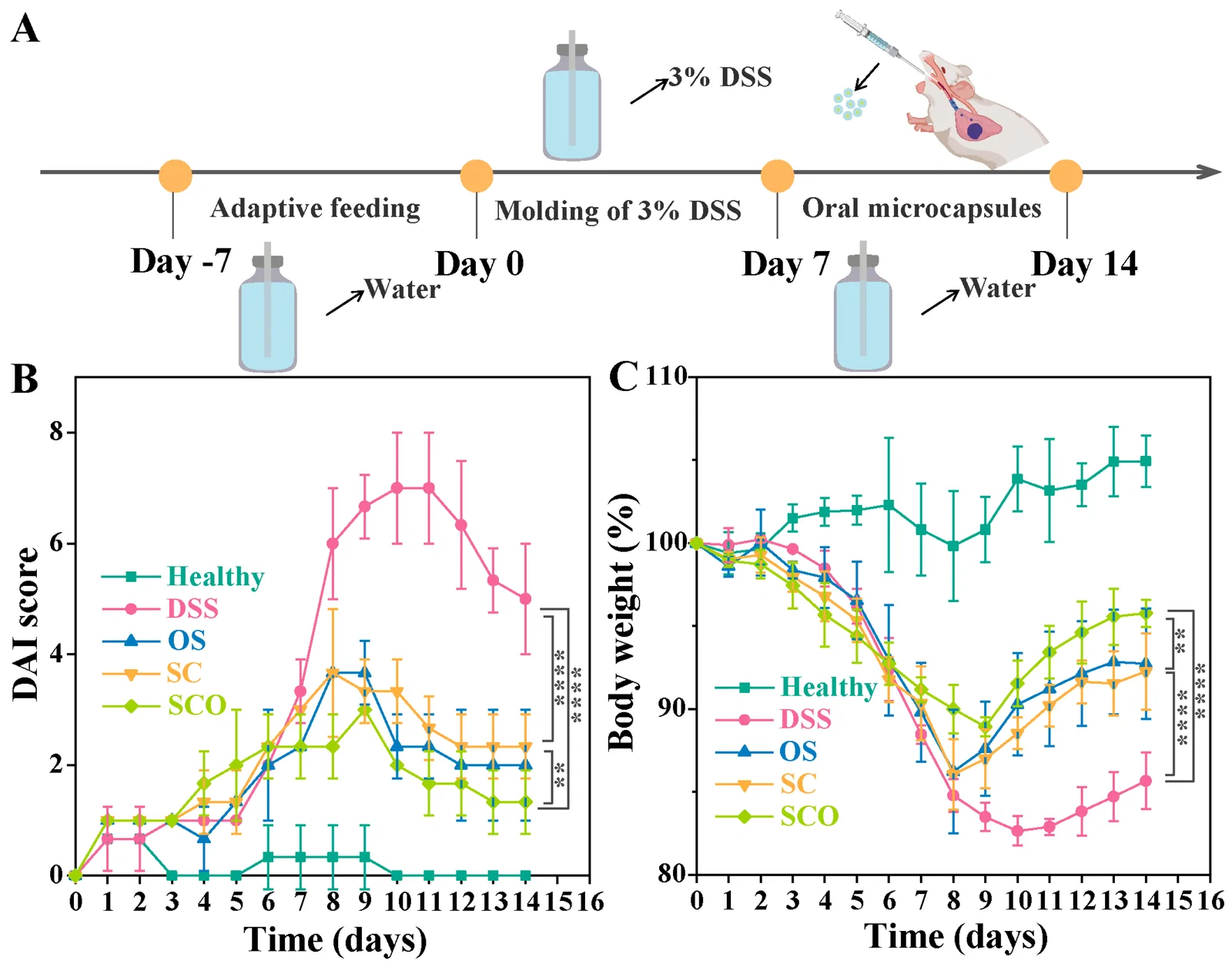
Schematic timeline and in vivo dynamic evaluation of SCO microcapsules in a DSS-induced ulcerative colitis (UC) mouse model. (**A**) Schematic workflow of the animal experiment: mice received adaptive feeding from Day −7 to Day 0, followed by administration of 3% DSS drinking water for UC establishment from Day 0 to Day 7 and oral administration of microcapsules from Day 7 to Day 14. (**B**) Time-dependent variations in disease activity index (DAI) scores of mice in the healthy, DSS, OS, SC, and SCO groups. (**C**) Dynamic changes in relative body weight (%) of mice from five experimental groups: healthy, DSS, OS, SC, and SCO groups. All data are expressed as mean ± SD; ** *p* < 0.01, **** *p* < 0.0001.

In this experiment, a well-studied model of UC pathology, DSS-induced acute colitis, was employed to assess the therapeutic efficacy of SCO microcapsules [63] (Figure 5A). During the experiment, mice with DSS-induced colitis were randomly assigned to five groups: a healthy control group, a DSS group, and three therapy groups (OS, SC microspheres, and SCO microcapsules). Figure 5C shows that during model induction, mice in the UC group lost weight more consistently than the mice in the healthy control group, especially the DSS group. Weight eventually recovered in all treatment groups after therapy, with the SCO group achieving the greatest weight restoration and a faster recovery rate than the positive-drug-control OS group.

DAI scores were used to evaluate UC severity and treatment efficacy by tracking body weight changes, stool consistency, and fecal blood from Days 0 to 14. The DAI score was determined following the classic protocol for DSS-induced colitis [64], by dividing the total sum of the weight loss score, stool consistency score, and hematochezia score by 3, with daily DAI data compiled in Table S1. Higher DAI scores indicate more severe UC (Table S2). The DAI score of the healthy control group only showed slight fluctuations at individual time points and stayed close to 0 throughout the experiment. In the 3% DSS-induced colitis model group, DAI scores began to increase from Day 1 after DSS administration. Overall, the DSS group had significantly higher DAI levels than the OS, SC, and SCO treatment groups over the whole treatment period (Figure 5B). In parallel, dynamic body weight changes were recorded throughout the experiment (Figure 5C), the DSS group suffered obvious continuous weight loss, while each microsphere treatment alleviated weight decline to different degrees, with the SCO group showing the best body weight recovery performance. After oral intervention with different microsphere formulations, DAI scores decreased to different extents in each treatment group. The OS and SC groups had much higher DAI values than the SCO group from Day 6 (Table S2). These results suggested that SCO microcapsules are more effective at treating UC than SC and OS when treatment time is extended, especially starting from the 6th day.

**Figure 6:**
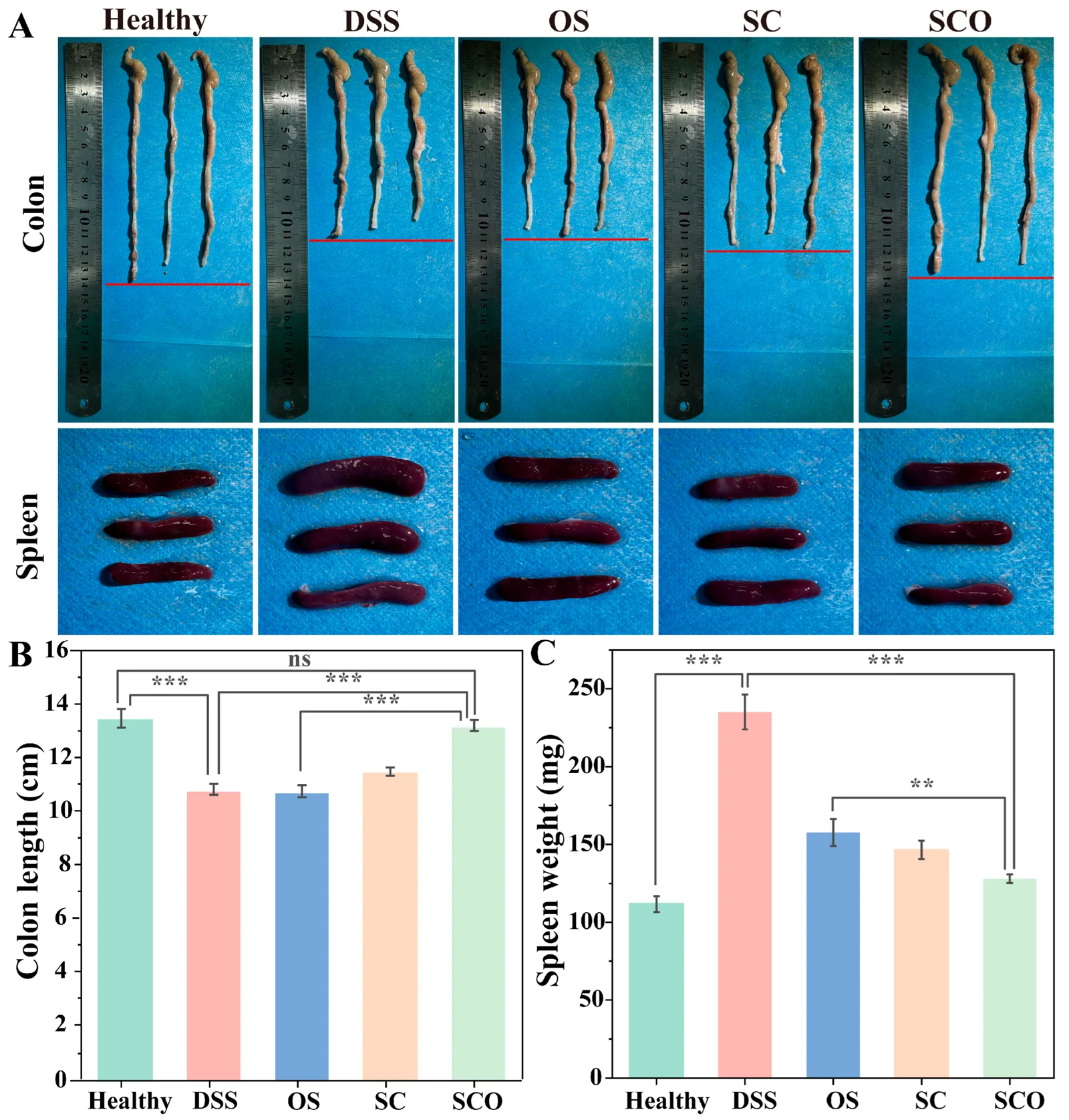
Macroscopic observation and quantitative analysis of colon and spleen tissues isolated from mice in healthy, DSS, OS, SC, and SCO groups. (**A**) Representative photographs of dissected colon and spleen tissues for healthy, DSS, OS, SC, and SCO groups. (**B**) Quantitative comparison of colon length of all groups. (**C**) Quantitative comparison of spleen weight across all groups. All data are presented as mean ± SD, ns represents no significant difference, ** *p* < 0.01, *** *p* < 0.001.

Colon length changes are a crucial pathophysiological indication in UC; a shorter colon corresponds to relatively more severe UC. Figure 6A shows the colon lengths for each group; the mean colon lengths of the DSS, OS, and SC groups were significantly shorter than that of the healthy group. After the UC mice were treated with SCO microcapsules, there was a dramatic recovery of colon length, which neared normal levels (Figure 6B). Spleen weight is also associated with the inflammatory response of UC mice. Figure 6A shows that the spleen weight in the treatment groups dramatically increased compared with the spleen weight in the healthy control mice, especially in the DSS groups, which had the highest spleen weight compared with other groups. Moreover, in the treatment group, the spleen weight of the SCO group was lower than the OS and SC groups (Figure 6C), almost approaching that of healthy mice, which indicated that SCO microcapsules have greater therapeutic effectiveness.

### 2.5. Histopathological Examination

In vivo biocompatibility was verified using H&E-stained slices of the major visceral organs (heart, lung, spleen, and kidney) isolated from the healthy mice and SCO microcapsule-treated mice, as illustrated in Figure S9. No remarkable pathological lesions, including tissue necrosis, severe inflammatory infiltration, or structural destruction, were found in the heart, spleen, and kidney between the SCO-microcapsule-treated mice and healthy mice. Only sporadic mild alveolar vacuolation was present in the lung of the SCO group without pathological significance. These histological results proved that SCO microcapsules possess satisfactory in vivo safety with negligible systemic organ toxicity.

**Figure 7:**
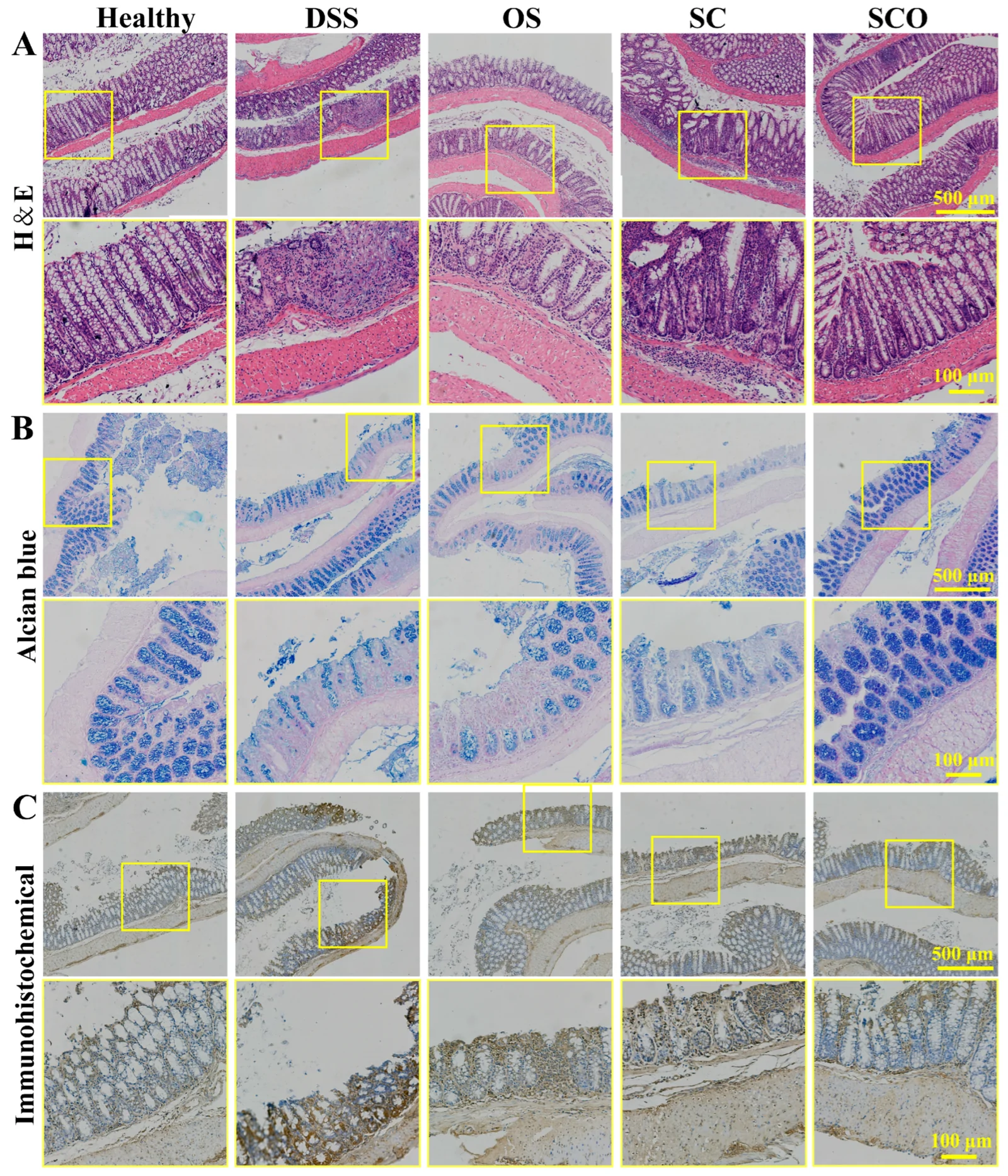
Representative histological images of colon tissues from healthy, DSS, OS, SC, and SCO groups. (**A**) Hematoxylin and eosin (H&E) staining for evaluation of colonic tissue inflammation and mucosal damage. (**B**) Alcian blue staining for goblet cells and intestinal mucin (blue color represents mucin). (**C**) Immunohistochemical staining of NF-*κ*B P65 (brown-yellow color indicates positive protein expression). Scale bars: upper panels 500 μm, lower panels 100 μm.

Then, the therapeutic efficiency of SCO microcapsules against DSS-triggered UC was systematically examined using a colon subject to H&E staining, Alcian blue staining, and NF-*κ*B immunohistochemistry (IHC) (Figure 7). After this, semiquantitative histopathological colonic injury scores were obtained, and quantitative statistical analyses of mucus-positive area and NF-κB p65-positive area were performed (Figure S10). The H&E staining (Figure 7A) showed that colonic tissues from the healthy group possessed regularly arranged intact crypts and minimal inflammatory infiltration within the lamina propria. The DSS group exhibited severe pathological destruction characterized by crypt atrophy/loss, mucosal erosion, and extensive inflammatory cell infiltration into the submucosa. The OS and SC groups displayed partial remission of colon injury with residual moderate inflammation and incomplete gland reconstruction. By comparison, the SCO group achieved prominent histological recovery, showing well-preserved crypt architecture and drastically alleviated inflammatory infiltration.

Alcian blue staining was further applied to visualize goblet cells and intestinal mucin (with positive signal presented as blue staining, Figure 7B). Abundant blue-stained mucin and an intact mucus barrier were observed in the healthy colon. DSS administration induced obvious goblet cell depletion and a sharp decline in mucin secretion, resulting in sparse faint blue signals (28.9%). Only limited mucin restoration was detected in the OS and SC groups, while SCO treatment markedly preserved the goblet cell population and mucus production (45.8%), restoring the mucus layer to a level comparable with the healthy group (43.8%).

IHC analysis of NF-*κ*B p65 was conducted to explore the underlying anti-inflammatory mechanism, with brown-yellow staining defined as positive protein expression (Figure 6C). Dramatically enhanced brown immunoreactivity was observed in the DSS group (38.6%), indicative of robust NF-*κ*B p65 overexpression and excessive inflammatory activation. Moderately reduced positive staining was found in the OS and SC groups, whereas SCO treatment significantly downregulated NF-*κ*B p65 expression, with 19.8% for the OS group and 16.1% for the SCO group, with the staining intensity approaching that of normal controls (20.8%).

Collectively, these histological findings demonstrated that SCO microcapsules ameliorate DSS-induced colitis by restoring colonic crypt structure, repairing the goblet-cell-originated intestinal mucus barrier, and suppressing NF-*κ*B p65-associated inflammatory cascades.

The development of pH-sensitive, core–shell, drug-loaded hydrogel microcapsules represents a notable advancement in targeted therapy for UC. The core–shell design successfully encapsulates therapeutic agents, enhancing colonic targeting efficiency and providing sustained release kinetics relative to conventional oral administration. Although the encapsulation efficiency of the microcapsule used in this experiment is modest, this value can be improved with a high concentration of Olsa during preparation. In vitro release studies demonstrated that the microcapsules retained minimal drug leakage under acidic conditions while facilitating rapid and controlled release at colonic pH (pH ≈ 7.4). This pH-responsive behavior is essential for protecting the drug from gastric degradation and preventing premature release in the upper gastrointestinal tract.

These findings are consistent with previous reports on pH-sensitive polymeric delivery systems [60,65], highlighting the potential of such formulations to improve drug stability during gastrointestinal transit. In vivo studies confirmed these advantages, showing significant reductions in inflammatory markers and histological damage scores in UC models treated with microcapsules compared with free drug administration and the mixed microsphere. The improved therapeutic outcomes can be attributed to precise drug localization in inflamed colonic tissue, which enhances bioavailability while minimizing systemic exposure and potential off-target toxicities. Moreover, the hydrogel network facilitates diffusion-controlled drug release. Despite these promising results, several challenges remain; batch-to-batch consistency and long-term safety are necessary before clinical application. Additionally, Tang et al. [66] reported that DSS treatment significantly reduced gut microbiota diversity. In contrast, the drug-loaded hydrogel group exhibited a curve most like that of the normal group, suggesting that this intervention effectively reversed DSS-induced dysbiosis and holds promise for restoring microbial diversity in UC mice. Therefore, the effects of repeated administration on gut microbiota composition and the potential for immune-mediated responses warrant further investigation.

In conclusion, this study established a robust framework for oral drug delivery systems targeting inflammatory bowel disease. By integrating pH-responsiveness with core–shell protection and controlled-release kinetics, these microcapsules have the potential to improve patient compliance and therapeutic outcomes in UC.

## 3. Conclusions

In this experiment, pH-sensitive core–shell hydrogel microcapsules were fabricated efficiently using SA and CMCS via electrostatic interactions and hydrogen bonding. These microcapsules were developed for targeted delivery to modulate UC intestinal inflammation. Utilizing the pH-sensitive features of SA and the biocompatibility of CMCS as the shell matrix enhanced the local bioavailability of the therapeutic agent. A UC mouse model demonstrated sustained and controlled drug release at the target site. Oral administration of the microcapsules significantly reduced NF-*κ*B p65 expression levels and substantially alleviated colitis-related pathological symptoms in a DSS-induced colitis mouse model (n = 3 per group). However, these in vivo observations are exploratory and statistically underpowered; the small sample size precludes definitive conclusions regarding therapeutic efficacy. The apparent biological effects require validation in adequately powered studies (n ≥ 6–8 per group) with rigorous blinding and randomization before clinical relevance can be asserted. Overall, this proof-of-concept study establishes the feasibility of core–shell microcapsules for pH-responsive colonic drug delivery with significant therapeutic potential for UC.

## 4. Materials and Methods

### 4.1. Materials and Reagents

Olsalazine sodium (Olsa, >99%) and sodium alginate (SA) were purchased from Aladdin (Shanghai, China). Carboxymethyl chitosan (CMCS, degree of substitution ≥90%) was purchased from McLean (Shanghai, China). Sodium dextran sulfate (DSS, molecular weight = 5000) was purchased from Heowns Biochem LLC (Tianjin, China). Citric acid (CA, analytical grade, ≥99.5%) was purchased from Beichen Fangzheng Reagent Factory (Tianjin, China). Sodium chloride (NaCl, analytical grade, ≥99.5%), hydrochloric acid (HCl, analytical grade, 36.0–38.0%), potassium dihydrogen phosphate (KH_2_PO_4_, analytical grade, ≥99.5%), and dipotassium hydrogen phosphate (anhydrous, K_2_HPO_4_, analytical grade, ≥99.0%) were all purchased from Sinopharm Chemical Reagent Co., Ltd. (Shanghai, China). Sodium bicarbonate (NaHCO_3_, analytical grade) was obtained from Tianjin Damao Chemical Reagent Partnership Enterprise (Limited Partnership) (Tianjin, China). Neutral buffered 4% paraformaldehyde fixative was obtained from Wuhan Servicebio Technology Co., Ltd. (Wuhan, China). L929 cells used in this experiment were purchased from Qidabio (Shanghai, China). Hematoxylin-Eosin (H&E) Stain Kit, 0.01 M phosphate-buffered saline (PBS) powder (pH 7.2–7.4), sodium citrate antigen retrieval solution, neutral balsam, DAB substrate kit, and Alcian Blue Periodic Acid Schiff (AB-PAS) Stain Kit were all purchased from Beijing Solarbio Science & Technology Co., Ltd. (Beijing, China).

### 4.2. Preparation of SA/CMCS Microspheres

In this study, SA/CMCS gel microspheres (SC) were prepared with a microencapsulation granulator (Encapsulator B-390, BÜCHI Labortechnik AG, Flawil, Switzerland) through physical crosslinking. Before the preparation of the microspheres, SA and CMCS were dissolved in deionized water, thoroughly stirred, and filtered to remove insoluble impurities, yielding concentrations of 1% SA and 2% CMCS (*w*/*v*) solutions, respectively. Then, the SA and CMCS stock solutions were mixed in the designed ratios and ultrasonically treated. A microencapsulation granulator with a single-channel nozzle connection was used as the microchannel. Increased air pressure facilitated dropwise injection into the CA solution (1% *w*/*v*), resulting in SC gel microspheres. The crosslinking reaction was carried out at room temperature for 1 h. Then, the SC gel microspheres were collected, washed several times with deionized water, and freeze-dried for future studies.

### 4.3. Preparation of SA/CMCS/Olsa Microcapsules

During the preparation of SA/CMCS/Olsa microcapsules, SA/CMCS mixed solutions at various ratios (3:1, 2:1, 1:1, 1:2, and 1:3) were used as the shell solutions, with Olsa dissolved in the SA/CMCS mixed solution serving as the core solution, and were named S_3_C_1_O, S_2_C_1_O, S_1_C_1_O, S_1_C_2_O, and S_1_C_3_O. A microencapsulation granulator equipped with a coaxial core–shell dual-channel nozzle was employed. Droplets were extruded under regulated air pressure into a 1% (*w*/*v*) CA solution to fabricate core–shell microcapsules. The crosslinking reaction in the 1% (*w*/*v*) solution proceeded at room temperature for 1 h. Then, the SCO microcapsules were collected, rinsed multiple times with deionized water, and freeze-dried for subsequent studies.

### 4.4. Characterization of the Microcapsules

The morphology of the microcapsules was characterized with a scanning electron microscope (SEM, Hitachi SU820, Hitachi High-Tech, Tokyo, Japan). The functional groups of the microcapsules were monitored using a Fourier-transform infrared spectrometer (FTIR, Nicolet iS10, Thermo Fisher Scientific, Waltham, MA, USA) with a scanning range of 400–4000 cm^−1^. Thermogravimetric analysis (TGA) of the microcapsules was conducted with a TGA instrument (STA2500, NETZSCH-Gerätebau GmbH, Selb, Germany) under a N_2_ atmosphere at a heating rate of 10 °C/min with a heating temperature range from 30 °C to 600 °C. The crystalline characteristics of the raw materials and the microcapsules were analyzed using X-ray diffraction (XRD) measurement (PANalytical Empyrean, Almelo, The Netherlands), employing Cu Kα radiation (λ = 1.54056 Å) over a 2*θ* range of 5° to 80°.

### 4.5. In Vitro Drug Release Measurement

To imitate the cumulative release of SCO microcapsules in the gastrointestinal (GI) tract, 20 mg of microcapsules was placed in a dialysis bag. The dialysis bag was immersed in a glass bottle containing 20 mL of simulated gastric fluid (SGF, pH = 1.2) and placed in a constant-temperature shaking incubator. A rotation speed of 100 rpm and a temperature of 37 ± 0.5 °C were maintained. After 2 h, the dialysis bag was transferred to a glass container with an equivalent volume of simulated intestinal fluid (SIF, pH = 6.8) and placed in a constant-temperature shaking incubator. After 3 h, the dialysis bag was transferred to a glass bottle filled with an equivalent volume of simulated colonic fluid (SCF, pH = 7.4) and placed in the constant-temperature shaking incubator. We withdrew 1.0 mL samples at regular intervals and replaced them with an equal volume of a new medium. A UV–visible spectrophotometer was used to determine drug concentration, compute cumulative drug release, and plot the Olsa cumulative release curve [60]. The cumulative release Q (%) was calculated as follows:

(1) $$Q\left(\%\right)=\frac{C_{i}\times V+\sum_{j=1}^{i-1}C_{j}\times V_{0}}{M}\times 100$$

where *V* and *V*_0_ represent the total volume of the release medium and the volume of the extracted sample at each time interval. *M* represents the total amount of the added drug, and *C_i_* represents the drug concentration measured in the release medium at each time interval *i*.

### 4.6. Drug Loading and Encapsulation Efficiency Measurement

The Olsa content in SCO microcapsules was calculated using the direct digestion method. A total of 20 mg of microcapsules was mixed with 20 mL of phosphate-buffered saline (PBS, pH = 7.4). The mixture was agitated on a constant-temperature shaker at 37 °C for 48 h before being ultrasonically disrupted to ensure complete drug release from the microcapsules. Following centrifugation, the supernatant was collected, and the Olsa concentration was determined using a UV–vis spectrophotometer based on its standard line [67]. The encapsulation efficiency (*EE*) and loading capacity (*LC*) were estimated using Equations (2) and (3).

(2) $$EE\left(\%\right)=\frac{Actual\;drug\;loading\;capacity}{Theoretical\;drug\;loading\;capacity}\times 100\%$$

(3) $$LC\left(\%\right)=\frac{Drug\;content\;within\;microcapsules}{Quality\;of\;microcapsules}\times 100\%$$

### 4.7. Cytotoxicity Assay

Cytotoxicity of the microcapsules was evaluated with L929 mouse fibroblasts, which were cultured in Dulbecco’s Modified Eagle Medium (DMEM) supplemented with 10% fetal bovine serum (FBS) and 1% penicillin-streptomycin solution and maintained at 37 °C in a humidified atmosphere containing 5% CO_2_. For in vitro assays, L929 cells in the logarithmic growth phase were seeded at appropriate densities into 96-well plates and incubated for 24 and 72 h. Cell proliferation and cytotoxicity were assessed using the CCK-8 Cell Proliferation and Cytotoxicity Assay kit based on the absorbance at 450 nm. Additionally, cells were stained with the Calcein-AM/PI Live/Dead Cell Double Stain Kit, and live/dead cell status was determined using an inverted fluorescence microscope (Eclipse E800, Nikon Corporation, Tokyo, Japan) [68].

### 4.8. Establishment of a DSS-Induced Ulcerative Colitis Model and Evaluation of Drug Therapy

All animal experiments were approved by the Institutional Animal Care and Use Committee (IACUC) of Lanzhou Veterinary Research Institute, Chinese Academy of Agricultural Sciences (Approval No.: LVRIAEC-2025-138). Male Balb/c mice (weighing 25–27 g, 8–12 weeks old) were acclimated for one week to familiarize them with their surroundings and ensure they achieved a stable state prior to further tests. Randomization was performed using a computer-generated random number sequence (Microsoft Excel RAND function) by an investigator not involved in subsequent data collection. Mice were assigned to five groups: healthy (control), DSS, OS (Olsa), SC, and SCO (n = 3). Group allocation was concealed in sequentially numbered, opaque, sealed envelopes until the day of induction. Induction phase: from days 0 to 7, the healthy group had free access to deionized water, whereas the other groups were induced with acute colitis by freely consuming 3% (*w*/*v*) dextran sulfate sodium (DSS). Treatment phase: from days 8 to 14, following colitis induction, the healthy group was given saline via gastric lavage; the OS, SC, and SCO groups were administered the same amount of Olsa, SC microspheres, or SCO microcapsules, respectively [69].

### 4.9. Clinical and Histological Evaluation of Ulcerative Colitis

From Day 1 to Day 14, daily measurements and records of mouse body weight, fecal consistency, and fecal hemorrhage were taken to track the disease activity index (DAI) [70].

(4) $$DAI=\frac{Body\;Weight\;Loss\;Score+Fecal\;Condition+Fecal\;Occult\;Blood}{3}$$

On Day 14, the mice were killed, and the important organs such as the colon and spleen were removed for further examination. Colon length and spleen weight were used as macroscopic markers of colitis severity. Then, the colon and organs were fixed in 4% formaldehyde solution, dried in alcohol, paraffin-embedded, sectioned at 5 μm thickness, and stained with hematoxylin and eosin (H&E) to analyze the histological characteristics [71]. Alcian blue staining was used to evaluate the histopathological characteristics of colon tissue [72]. Immunohistochemical analysis was conducted to assess intestinal epithelial barrier repair through the detection of NF-*κ*B (p65) expression [73]. Stained tissue sections were subsequently examined by light microscopy.

**Scheme 1:**
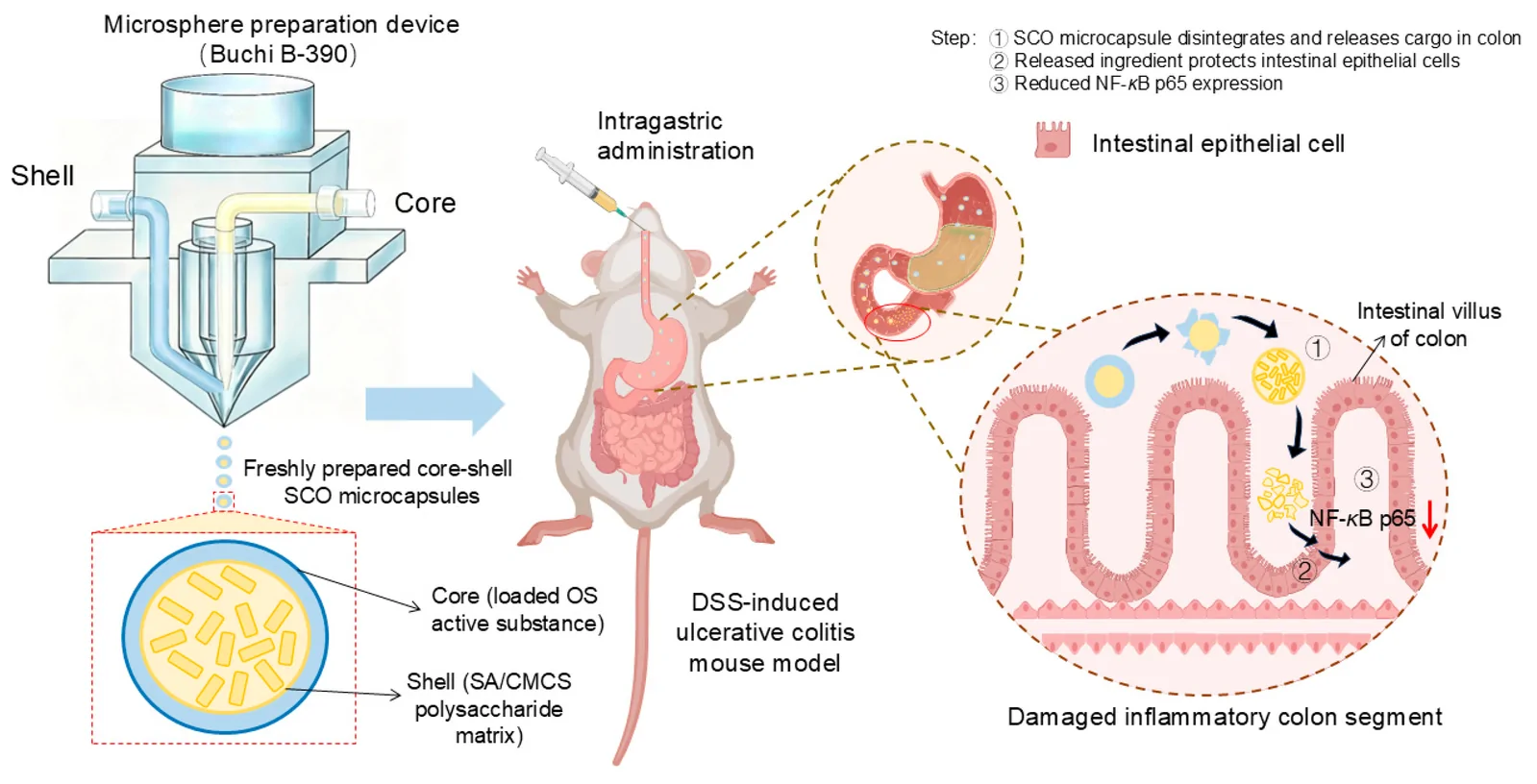
Schematic workflow for the preparation of pH-responsive SA/CMCS/Olsa core–shell microcapsules and in vivo therapeutic evaluation in DSS-induced ulcerative colitis mice: (1) fabrication of microcapsules via electrostatic microencapsulation using a Buchi B-390 microencapsulation device (BÜCHI Labortechnik AG, Switzerland); (2) oral gavage of microcapsules to mice with DSS-induced ulcerative colitis; (3) collection of colonic tissue for histological staining and molecular biological analysis, the red downward arrow indicates the downregulation of NF-κB p65 expression. (DSS: dextran sulfate sodium; SA: sodium alginate; CMCS: carboxymethyl chitosan.)

The experimental workflow for the preparation and in vivo evaluation of pH-responsive SCO microcapsules is illustrated in Scheme 1.

### 4.10. Statistical Analysis

All results were expressed as the mean ± SD (standard deviation) from at least three tests. To assess statistical significance, one-way analysis of variance (ANOVA) and the Tukey test by Origin (Version 2024) were utilized, and the significance levels were indicated as follows: * *p* < 0.05, ** *p* < 0.01, *** *p* < 0.001, **** *p* < 0.0001, “ns” means no significant difference.

## Figures and Tables

**Table 1:** Kinetic release model expressing fitted drug release data from SCO microcapsules.

Constants	Korsmeyer−Peppas	Higuchi	Hixson−Crowell	First-Order	Zero-Order
Regression coefficient (*R*^2^)	0.764	0.817	0.714	0.805	0.637
Diffusion exponent (*n*)	0.51	-	-	-	-

## Data Availability

The data presented in this study are available on request from the corresponding author.

## Supplementary Materials

The following supporting information can be downloaded at https://www.mdpi.com/article/10.3390/gels12080718/s1, Figure S1: Optical microscopy images of SC microspheres and core–shell SCO microcapsules. The red circles highlight the distinct core structure of SCO microcapsules. Scale bar: 500 μm; Figure S2: Fluorescence microscopy image of SCO microcapsules. Scale bar: 500 μm; Figure S3: (A) The size distribution of dry SA microspheres and (B) the size distribution of SCO microcapsules; Figure S4: The standard release curves for olsalazine sodium at (A) pH 1.2, (B) pH 6.8, and (C) pH 7.4, respectively; Figure S5: The microscopy images (A) and SEM results (B) of the microcapsules prepared with different ratios of SA and CMCS, including S_3_C_1_O, S_2_C_1_O, S_1_C_1_O, S_1_C_2_O, and S_1_C_3_O; Figure S6: (A) Fourier-transform infrared (FT-IR) spectra of SA, CMCS, and SCO microcapsules with varied SA/CMCS ratios (S_3_C_1_O, S_2_C_1_O, S_1_C_1_O, S_1_C_2_O, and S_1_C_3_O). (B) Thermogravimetric analysis (TGA) curves of SA, CMCS, and SCO microcapsules (S_3_C_1_O, S_2_C_1_O, S_1_C_1_O, S_1_C_2_O, and S_1_C_3_O); Figure S7: Time-dependent swelling behavior of different hydrogel microspheres. (A) Swelling profiles measured in simulated gastric fluid (pH = 1.2). (B) Swelling profiles measured in simulated intestinal fluid (pH = 7.4). S_3_C_2_, S_2_C_1_, S_1_C_1_, S_1_C_2_, and S_1_C_3_ represent hydrogel microspheres with different ratios of SA and CMCS; Figure S8: Kinetic equations fit to different models (Equations (S1)–(S5)), reflecting Olsa release from the S_2_C_1_O microcapsules in a pH 6.8~7.4 solution; Figure S9: H&E staining of major organ tissues, including the heart, lung, spleen, and kidney in SCO-microcapsule-treated mice and the healthy group mice; Figure S10: (A) Semiquantitative histopathological injury score of colon tissues from each experimental group. (B) Quantitative statistical analysis of mucus-positive area. (C) Quantitative statistical analysis of NF-*κ*B p65-positive area. ** *p* < 0.01, *** *p* < 0.001. Table S1: Daily DAI scores of mice in each experimental group; Table S2: Detailed scoring criteria for the DAI activity index.
