# Supplementary material for: Efficient Preparation of pH-Sensitive Core–Shell Drug-Loaded Hydrogel Microcapsules and Their Application in Ulcerative Colitis Treatment
Source: Gels. 2026 Aug 13;12(8):718. doi: 10.3390/gels12080718 (PMC13512429; doi:10.3390/gels12080718)
Supplement: Supplementary file 1 [file gels-12-00718-s001.zip › gels-4426325-supplementary.pdf]

Supporting Information

Efficient Preparation of pH-Sensitive Core-Shell Drug-Loaded Hydrogel Microcapsules and Their Application in Ulcerative Colitis Treatment

Qingqing Xue [1, 2], Yingli Li [1, 3], Qing Ao [1, 2], Guowang Chang [1,2], Yang Ji [4], Shizhang Chen [4], Ze Wang [1, 3], Zifan Wang[1, 2], Zhiqiang Li [5], Lei Zhao [1, 2 *]

1 Key Laboratory of Biotechnology and Bioengineering of State Ethnic Affairs Commission, Biomedical Research Center, Engineering Research Center of Key Technology and Industrialization of Cell-based Vaccine, Ministry of Education, Gansu Tech Innovation Center of Animal Cell, China-Malaysia National Joint Laboratory, Northwest Minzu University, Lanzhou 730030, China

2 School of Bioengineering College, Northwest Minzu University, Lanzhou 730030, China

3 College of Chemical Engineering, Northwest Minzu University, Lanzhou 730124, China

4 School of Life Sciences and Engineering, Northwest Minzu University, Lanzhou 730124, China

5 Department of Medical, Northwest Minzu University, Lanzhou 730030, China

* Correspondence: zhaolei@xbmu.edu.cn (L.Z.)

1. Core-shell Structure of SCO microcapsules

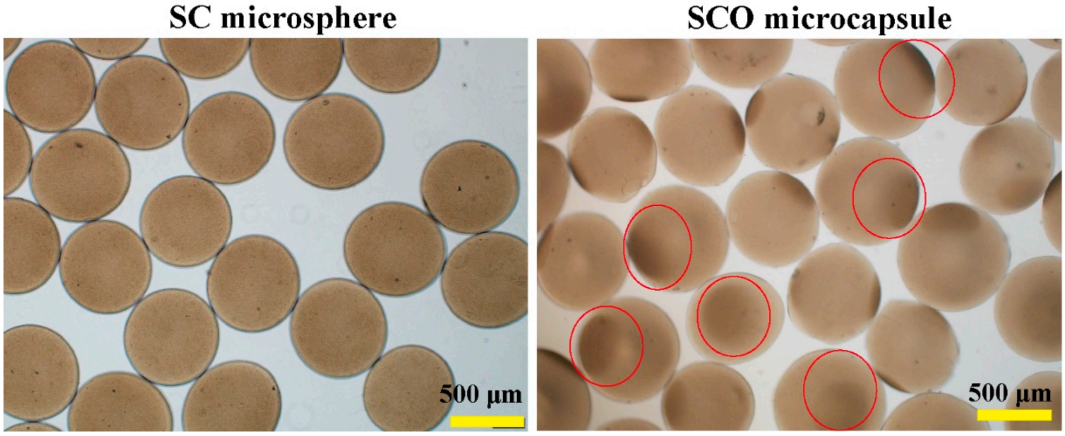

Figure S1. Optical microscopy images of SC microspheres and core-shell SCO microcapsules. The red circles highlight the distinct core structure of SCO microcapsules. Scale bar: 500 μm.

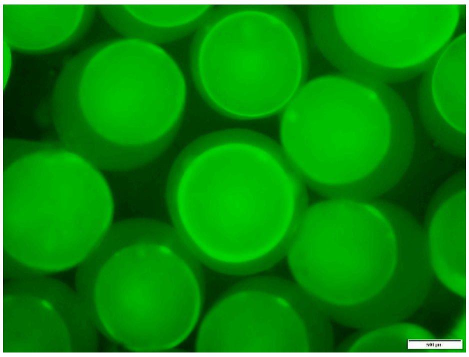

Figure S2. Fluorescence microscopy image of SCO microcapsules. Scale bar: 500 μm.

2. Particle Size Distribution of SA Microspheres and SCO Microcapsules

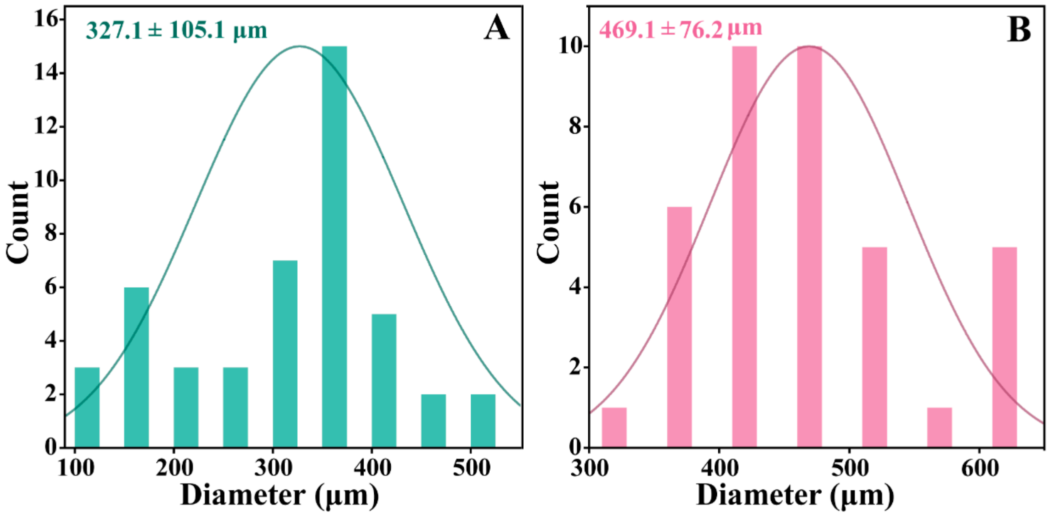

Figure S3. A The size distribution of dry SA microspheres and **B** the size distribution of SCO microcapsules.

3. Standard Calibration Curves of Olsalazine Sodium at Different pH Values

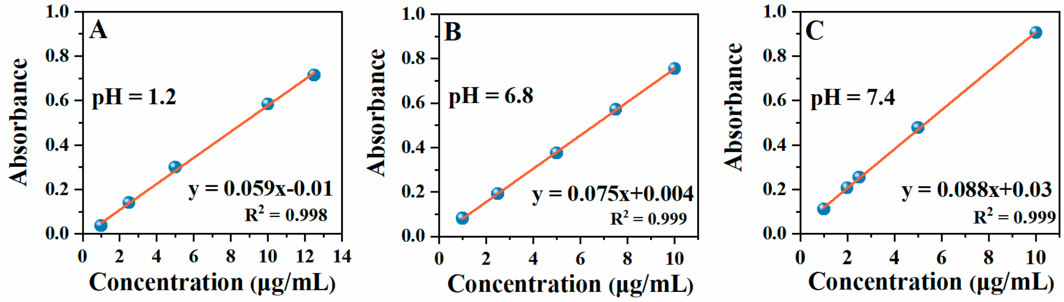

Figure S4. The standard release curves for olsalazine sodium at (**A**) pH 1.2, (**B**) pH 6.8, and (**C**) pH 7.4, respectively.

4. Optimization of the Preparation of SCO Microcapsules

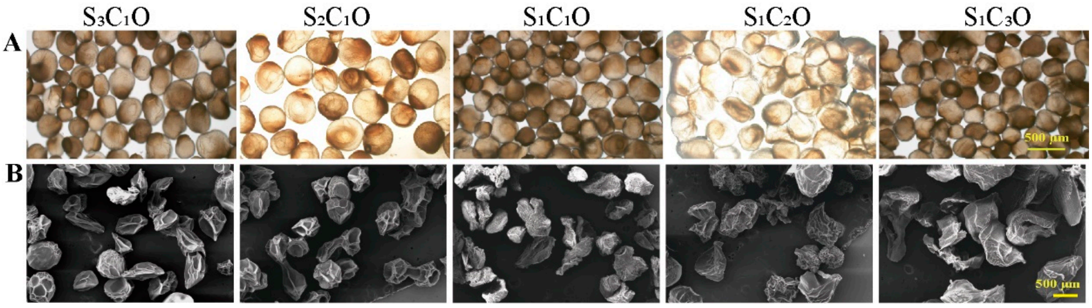

Figure S5. The microscopy images (**A**) and SEM results (**B**) of the microcapsules prepared with different ratios of SA and CMCS, including S_3C_1O, S_2C_1O, S_1C_1O, S_1C_2O, and S_1C_3O.

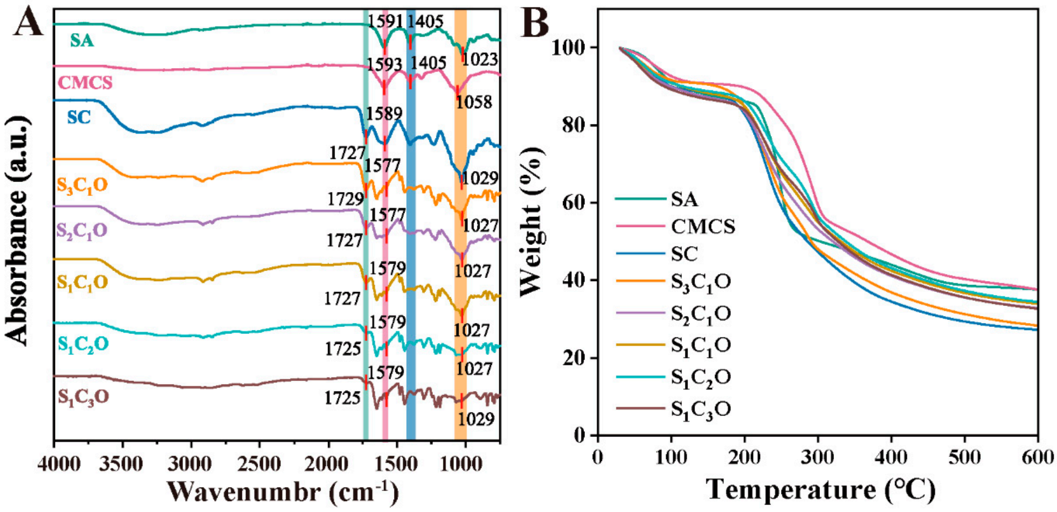

Figure S6. (**A**) Fourier-transform infrared (FT-IR) spectra of SA, CMCS, and SCO microcapsules with varied SA/CMCS ratios (S_3C_1O, S_2C_1O, S_1C_1O, S_1C_2O and S_1C_3O). (**B**) Thermogravimetric analysis (TGA) curves of SA, CMCS and SCO microcapsules (S_3C_1O, S_2C_1O, S_1C_1O, S_1C_2O and

S_1C_3O).

5. Swelling Ratio of SC Microspheres at Different Ratios of SA/CMCS

At pH 1.2 (Figure S7A), rapid water absorption occurred within the first 30 min, followed by a slow increase until swelling equilibrium was reached. The final swelling ratio remained at a relatively low level under acidic conditions. Siginificant differences in equilibrium swelling capacity were observed among groups with different SA/CMCS ratios. The S_2C_1 microsphere displayed the lowest swelling degree with 18.0% at 3 h.

At pH 7.4 (Figure S7B), all microspheres achieved a much higher swelling ratio compared with that in simulated gastric fluid. A similar swelling profile was observed with an initial fast-swelling stage and a subsequent plateau. The equilibrium swelling ratio varied with the CMCS proportion, and the S_2C_1 sample exhibited the maximum swelling capacity as 4090.3% at 7 h.

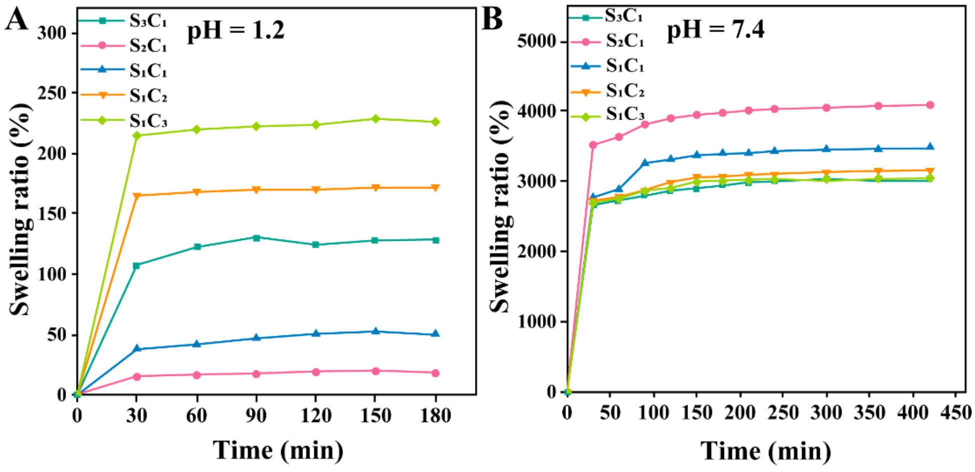

Figure S7. Time-dependent swelling behavior of different hydrogel microspheres. (**A**) Swelling profiles measured in gastric simulated fluid (pH = 1.2); (**B**) Swelling profiles measured in intestinal simulated fluid (pH = 7.4). S_3C_2, S_2C_1, S_1C_1, S_1C_2, S_1C_3 represent hydrogel microspheres with different ratio of SA and CMCS.

6. The Release Kinetics of SCO Microcapsules

According to the review literature, there are different types of drug release kinetics and mechanisms based on the polymer matrix. Recently, the most probable kinetics have been used for drug release[1], as depicted below:

Zero-order kinetics

$$W_t = W_0 + k_1 t \qquad \text{(S1)}$$

First-order kinetics

$$logC_t = logC_0 - k_2 t/2.303 \qquad \text{(S2)}$$

Hixson-Crowell's cube-root equation (erosion model)

$$(100 - W)^{1/3} = 100^{1/3} - k_3 t \qquad \text{(S3)}$$

Higuchi's square root of time equation (diffusion model)

$$W_t = k_4 t^{1/2} \qquad \text{(S4)}$$

Korsmeyer-Peppas (diffusion/relaxation model)

$$M_t/M_\infty = k_5 t^n \qquad \text{(S5)}$$

where *k* is the drug release constant; C_t and C_0 are the concentrations of drug at the initial time and testing time; W_t and W_0 are the weights of the drug at the initial time and testing time; M_t/M_∞ is the fractional drug release into the dissolution medium; and *n* is the diffusional constant that characterizes the drug release transport mechanism.

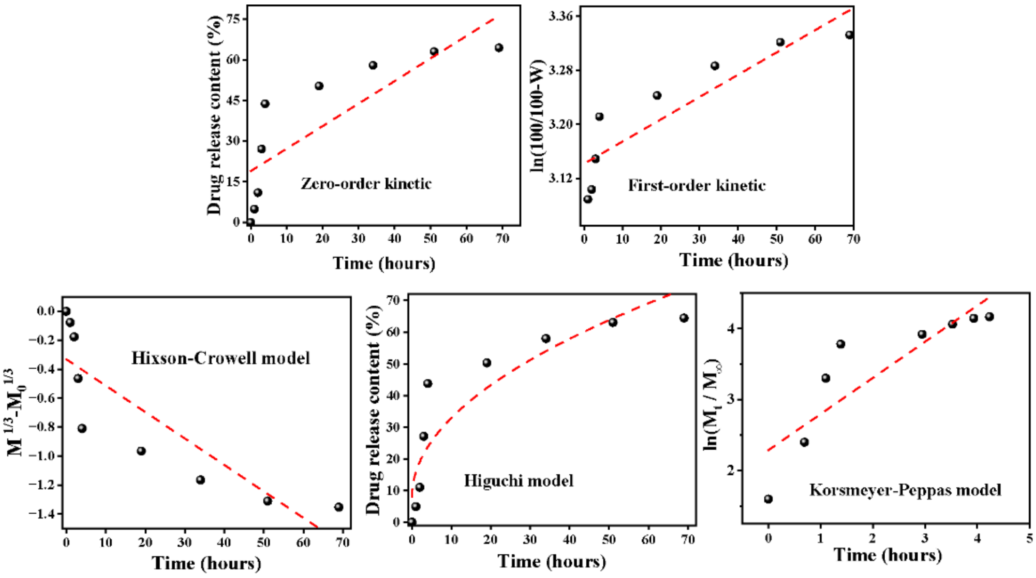

Figure S8. Kinetic equations fit to different models (Equation s1~ s5), reflecting Olsa release from the S_2C_1O microcapsules in pH 6.8 ~ 7.4 solution.

7. *In Vivo* Therapeutic Efficacy of SCO Microcapsules on DSS-Induced Colitis Mice

Table S1. Daily DAI scores of mice in each experimental group.

Day	Healthy	DSS	OS	SC	SCO
D0	0	0	0	0	0
D1	0.67 ± 0.58	0.67 ± 0.58	1	1	1
D2	0.67 ± 0.58	0.67 ± 0.58	1	1	1
D3	0	1	1	1	1
D4	0	1	0.67 ± 0.58	1.33 ± 0.58 [a]	1.67 ± 0.58 [a]
D5	0	1	1.33 ± 0.58	1.33 ± 0.58	2 ± 1 [a]
D6	0.33 ± 0.58	2	2 ± 1	2.33 ±0.58 [a]	2.33 ± 0.58 [a]
D7	0.33 ± 0.58	3.33 ± 0.58 [a]	2.33 ± 0.58 [a]	3 [a]	2.33 ± 0.58 [a]
D8	0.33 ± 0.58	6 ± 1 [a]	3.67 ± 1.15 [a]	3.67 ± 1.15 [a]	2.33 ±0.58 [b]
D9	0.33 ± 0.58	6.67 ± 0.58 [a]	3.67 ± 0.58 [ab]	3.33 ± 0.58 [ab]	3 [ab]
D10	0	7 ± 1 [a]	2.33 ± 0.58 [ab]	3.33 ± 0.58 [ab]	2 [ab]
D11	0	7 ± 1 [a]	2.33 ± 0.58 [ab]	2.67 ± 0.58 [ab]	1.67 ± 0.58 [b]
D12	0	6.33 ± 1.15 [a]	2 ± 1 [b]	2.33 ± 0.58 [ab]	1.67 ± 0.58 [b]
D13	0	5.33 ± 0.58 [a]	2 ± 1 [ab]	2.33 ± 0.58 [ab]	1.33 ± 0.58 [b]
D14	0	5 ± 1 [a]	2 ± 1 [ab]	2.33 ± 0.58 [ab]	1.33 ± 0.58 [b]

Values are presented as mean ± SD. [a] Significant difference at $P < 0.05$ compared with Healthy group; [b] Significant difference at $P < 0.05$ compared with DSS group.

Table S2. Detailed scoring criteria for DAI activity index.

Score value	Weight loss (%)	Fecal condition	Fecal occult blood
0	0	Normal	Negative
1	1-5	Soft stool	Light Blue
2	6-10	Mucus-like stool	Blue
3	11-15	Watery stool	Dark blue
4	> 15	/	Visible blood in stool

8. Histopathological Analysis of Major Organs in Mice

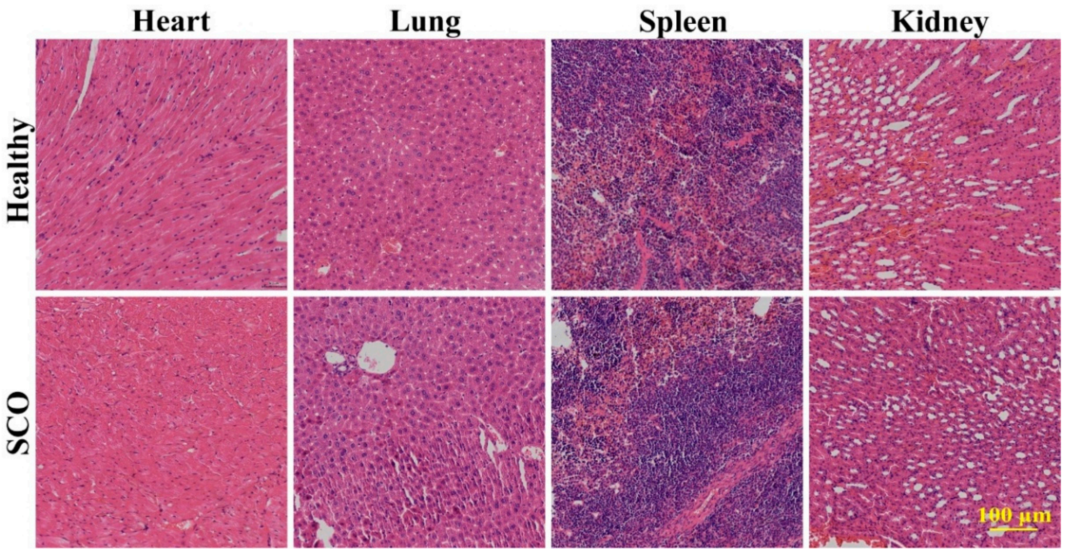

Figure S9. H&E staining of major organ tissues, including heart, lung, spleen, and kidney in SCO microcapsule treated mice and the healthy group mice.

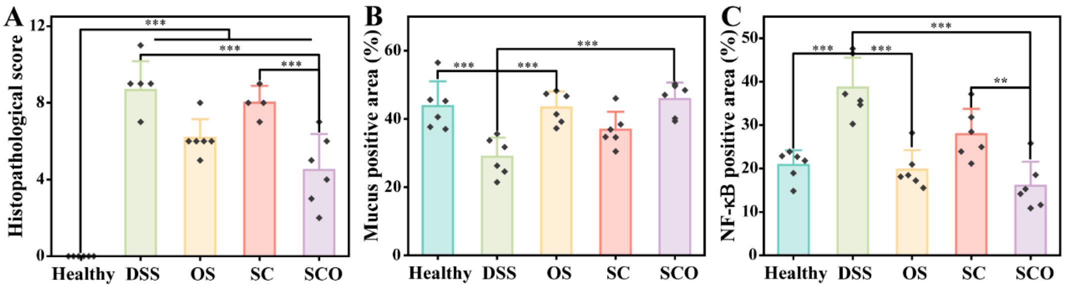

Figure S10. (**A**) Semiquantitative histopathological injury score of colon tissues from each experimental group; (**B**) Quantitative statistical analysis of mucus-positive area; (**C**) Quantitative statistical analysis of NF-κB p65 positive area. $^{**}p < 0.01$, $^{***}p < 0.001$.

Reference

1. Thai, H.; Thuy Nguyen, C.; Thi Thach, L.; Thi Tran, M.; Duc Mai, H.; Thi Thu Nguyen, T.; Duc Le, G.; Van Can, M.; Dai Tran, L.; Long Bach, G.; et al. Characterization of Chitosan/Alginate/Lovastatin Nanoparticles and Investigation of Their Toxic Effects in *Vitro* and in *Vivo*. *Sci. Rep.* **2020**, *10*, 909–924, doi: 10.1038/s41598-020-57666-8.
